# Using institutional ethnography to analyse animal sheltering and protection II: Animal shelter work

**DOI:** 10.1017/awf.2023.83

**Published:** 2023-10-03

**Authors:** Katherine E Koralesky, Janet M Rankin, David Fraser

**Affiliations:** 1 Animal Welfare Program, University of British Columbia, 2357 Main Mall, Vancouver, BC V6T1Z4, Canada; 2 Faculty of Nursing, University of Calgary, 2500 University Drive NW Calgary, AB T2N1N4, Canada

**Keywords:** animal welfare, behaviour, dogs, employee, length of stay, qualitative

## Abstract

Efficient adoption is an important aim of animal shelters, but it is not possible for all animals including those with serious behavioural problems. We used institutional ethnography to explore the everyday work of frontline shelter staff in a large animal sheltering and protection organisation and to examine how their work is organised by standardised institutional procedures. Shelter staff routinely conduct behavioural evaluations of dogs and review intake documents, in part to plan care for animals and inform potential adopters about animal characteristics as well as protect volunteers and community members from human-directed aggression. Staff were challenged and felt pressure, however, to find time to work with animals identified as having behavioural problems because much of their work is directed toward other goals such as facilitating efficient adoption for the majority and anticipating future demands for kennel space. This work is organised by management approaches that broadly aim to maintain a manageable shelter animal population based on available resources, decrease the length of time animals spend in shelters and house animals based on individual needs. However, this organisation limits the ability of staff to work closely with long-stay animals whose behavioural problems require modification and management. This also creates stress for staff who care for these animals and are emotionally invested in them. Further inquiry and improvements might involve supporting the work of behavioural modification and management where it is needed and expanding fostering programmes for animals with special needs.

## Introduction

Efficient adoption is a central aim of animal shelters, but this is not feasible for all animals. First, behavioural problems are a major reason for the relinquishment of animals to shelters (Weiss *et al.* 2015; Powell *et al.* 2021). Further, animals with longer shelter stays are more likely to become ill or develop a behavioural problem (Protopopova 2016; Wagner *et al*. 2018) which can make adoption a more difficult and lengthy process. For example, dogs with behavioural problems such as aggression and high arousal had longer shelter stays (Raudies *et al.* 2021). Extremely fearful dogs can also have longer stays, however, Collins *et al.* (2022) reported a 99% adoption rate for fearful dogs that completed a behavioural rehabilitation programme over a two-month period. Furthermore, shelter facilities have been designed for short-term stays and do not adequately provide for animals’ long-term physical and behavioural needs (Association of Shelter Veterinarians 2022).

Noting the problems of long stays in shelters, researchers have aimed to identify risk factors for length of stay (LOS), a time-based measure, usually by analysing relationships between average LOS and animal characteristics such as age, sex, breed and coat colour (Brown *et al.* 2013; Patronek & Crowe 2018; Voslářová *et al.* 2019). The goal of such research is to determine how shelters can promote adoption of certain animals (e.g. older animals), better understand adopter preferences and prevent euthanasia by adopting animals more quickly (Koralesky *et al.* 2023a). Such research typically relies upon categories of animals and calculates measures of central tendency (e.g. mean, median) to report LOS.

Achieving a short LOS for shelter animals has not always been a central focus in animal sheltering. In North America in the 1970s, concern about pet ‘overpopulation’ (i.e. when the number of stray or unwanted animals exceeded the number of potential adopters) and high rates of euthanasia led veterinary and humane organisations to explore how to manage the problem (Rowan & Wilson 1985; Salman *et al.* 1998). These organisations implemented a multi-pronged approach of legislation (e.g. licensing requirements for dogs), owner education and pet sterilisation to decrease overpopulation. Over time, these measures, plus increases in adoption rates and trap-neuter-return programmes (Levy *et al.* 2014), greatly decreased the number of animals euthanased in shelters (Protopopova 2016; Rowan & Kartal 2018; Humane Canada 2022; American Society for the Prevention of Cruelty to Animals [ASPCA] 2023).

Approximately ten years ago, Capacity for Care (C4C) was developed as a shelter population management programme in North America that calculates the optimal shelter animal population that can be provided with humane care based upon available resources and other factors (Koret Shelter Medicine Program 2016; BC SPCA 2022). A key idea of C4C is that by maintaining an optimal population, animal outcomes are optimised by reducing euthanasia and increasing adoptions (Koret Shelter Medicine Program 2016). Shelters that operate beyond their capacity are unable to provide adequate care for all animals, compromising welfare and increasing LOS (Association of Shelter Veterinarians 2022). Research about C4C has focused on animal (cats) outcomes. For example, Janke *et al.* (2017) and Karsten *et al.* (2017) statistically analysed shelter databases and reported that the use of C4C decreased average LOS to adoption for cats; Janke *et al.* (2018) reported that C4C decreased the number of cats brought to the shelter; and Hobson *et al.* (2021a) investigated outcomes for cats that had been put on a waiting list (following C4C deferred intake) before a shelter would accept them. Thus, overall, this research uses measures of central tendency, broad categories of animals, time-based measures like LOS, and presents findings as metrics that demonstrate that C4C is working efficiently to manage cat populations in shelters.

What is less well understood, however, is how the everyday work of frontline shelter staff is connected to institutionalised processes and policies linked to C4C and specifically with animals that have behavioural or veterinary problems. These exceptional animals can spend long periods in shelters and are becoming more common in shelters today (Koralesky *et al.* 2023a). This paper presents the case of Henry, a dog who spent 60 days in a shelter and was eventually euthanased – the opposite outcome of what animal shelters aim to achieve for healthy animals that do not pose a risk to public safety. We use Henry’s story as an entry-point into the everyday work processes of an animal sheltering organisation that uses C4C. We do not dismiss the benefits of efficient shelter management but show how the well-being of an animal with a long LOS, and the concerns that frontline shelter staff have for these animals, can be at odds with institutional processes and priorities that achieve timely adoption of most animals.

## Materials and methods

### Ethical approval

The University of British Columbia Behavioural Research Ethics Board (#H19-00009) and the British Columbia Society for the Prevention of Cruelty to Animals (BC SPCA) approved this study. Animal handling and care was carried out by the BC SPCA in accordance with BC SPCA research guidelines.

Detailed methods are described by Koralesky *et al.* (2023b) and are outlined briefly below.

### Institutional ethnography

For this study we used institutional ethnography (IE), a form of inquiry that aims to discover the actual, everyday work of people in an institution and examine how these work activities are organised by institutional processes (Smith 1987, 1990, 2005). Through describing people’s everyday work, and examining how that work is co-ordinated with the work of other people (Smith 2005; p 52, 227), the aim of IE is to describe occasions when institutional aims (such as efficient adoption and low LOS for the majority of animals) and the everyday work of the people (in this case animal shelter staff and the animals they care for) result in tensions that are not captured in metric data. In IE, such occasions become a focus for analysis (Smith 2005; p 38).

Animal sheltering involves many individuals including frontline staff, animal protection officers (i.e. staff authorised to enforce animal protection laws, hereafter ‘officers’), administrators (including managers, veterinarians, behaviourists, etc.) and animals. Each individual’s experiences of institutional practices are co-ordinated in relation to where that individual is located within the institution (Smith 1987; p 107). In IE, the researcher adopts a “standpoint” location as a “point of entry” into inquiry (Smith 1990; p 5, p 10). We began inquiry from the standpoint of animals who have become involved with animal sheltering, although of course, human participants, in particular frontline shelter staff, were also critically important informants.

Taking the standpoint of animals involved observing them and what they were doing in their kennels and recording fieldnotes about how they communicated behaviourally (e.g. watching, growling, sniffing). We also observed frontline staff who had knowledge about animals including their biological health (by recording and reporting signs of illness such as coughing, sneezing, irregular urination and defaecation) and behaviours (by observing and recording behavioural signs of fear, anxiety, frustration and depression such as hiding, barking, lunging, jumping, whining, and also positive behavioural signs such as eating, grooming and playing). Our observations and discussions with frontline staff enhanced our understanding of the animals’ standpoint.

In this paper, we describe the everyday work of frontline shelter staff to discover how institutionalised processes and policies linked to C4C influence everyday practices. We explore a series of tensions that arise when frontline shelter staff manage animals with behavioural problems, like Henry, that complicate pathways to adoption.

### Research participants

This research is part of a larger project for which the BC SPCA was the central research partner. The BC SPCA is a large organisation (close to 600 staff and 4,000 volunteers) with 34 shelters, four hospitals and clinics, a wildlife rehabilitation centre and humane education programmes. The shelters vary in size but typically include animal care and customer service frontline staff, administrators and in some cases veterinary technicians. Before the COVID-19 pandemic began, in 2019 the BC SPCA reported mean LOS for groups of animals including dogs (ten days) and cats (15 days; BC SPCA 2020), compared to the national average of 24 days for dogs and 29 days for cats (Humane Canada 2020). These data do not include time spent in foster homes. Across Canadian shelters in 2019 (including BC SPCA shelters), an estimated 78,000 cats and under 28,000 dogs were taken into shelters (Humane Canada 2020); BC SPCA shelters had an intake of 5,322 dogs and 12,584 cats (BC SPCA 2022).

BC SPCA staff (administrators, managers, officers and frontline animal shelter staff), as well as non-human animals, were research participants. The primary author (KEK) is a long-serving volunteer with the organisation and has also volunteered in animal shelters in the USA. Before the study began, we met with frontline shelter staff, officers and administrators to discuss the study and answer questions.

We used the ethnographic methods of participant and naturalistic observation, interviews, focus groups and document analysis (Campbell & Gregor 2002; DeVault & McCoy 2006) for eight months in 2019; follow-up interviews were conducted as needed via telephone or virtually (Zoom Video Communications Inc, San Jose, CA, USA 2021) in 2020 after the COVID-19 pandemic began. As is typical in IE studies, our first focus was on frontline shelter staff because it is their work that typically connects clients (in this case, animals) to institutional texts and procedures (DeVault & McCoy 2006; p 27). Our analysis of the organisation of frontline workers (henceforth called staff) generated some of the questions we asked in our later conversations with administrators, officers and managers.

### Observations, interviews and document analysis

After staff provided written consent to participate in the research, KEK observed staff and made written fieldnotes as they performed their daily work activities in the shelter. When observations involved members of the public visiting the shelter, KEK briefly explained that she was a student researcher, asked if she could observe the interaction between the person and staff, and proceeded only after verbal consent had been given. Verbal consent is permitted by the Tri-Council Policy Statement on the Ethical Conduct for Research Involving Humans (TCPS 2 2022). Such observations are considered to be “minimal risk” because they are fieldwork practices and do not identify participants in the dissemination of results, are not covert, are not staged by the researcher and are non-intrusive (TCPS2, article 10.3, 2022; p 191–193).

During and after observing staff, KEK conducted interviews informally, asking staff for explanations about what they were doing, why, and how they used physical and digital texts (e.g. checklists, evaluation forms, the digital shelter database, laws). Texts are a central part of IE research because they organise what people do and how they communicate and co-ordinate their work (Smith & Turner 2014; p 5). During interviews, KEK asked follow-up questions about the work staff did, how they brought texts into their work activities, and co-ordinated their work with others. To protect participant confidentiality, all names are pseudonyms, the pronoun ‘they’ is used, and we altered certain data (e.g. locations, dates, number of animals involved in cases) in a way that maintains the approximate features of events without compromising confidentiality.

### Focus groups

We conducted four virtual focus groups on Zoom video communications software in early 2021 with BC SPCA personnel. Detailed methods are presented in Koralesky *et al.* (2023a). Briefly, the BC SPCA assisted with participant recruitment by sending a letter of invitation to all staff involved with animal management. We then held two focus groups with shelter staff (n = 2 and n = 4), one with officers (n = 5) and one with administrators (n = 11). We included individuals working across the organisation to identify connections between everyday work processes and to inquire into the role of texts in co-ordinating work processes across the organisation. Part of the focus groups involved asking participants to discuss how their everyday work experiences were represented in common research topics in animal sheltering, including LOS. Participants also shared specific work experiences that they categorised within the topics identified in the literature review.

### Data analysis

During data collection, we noted instances that seemed to cause tension, for example, when problems, frustrations or stress arose for people we observed and the animals they cared for. Events surrounding a dog named Henry are an example of such tensions, and thus we use his story as an entry-point to explore how those experiences arose.

We followed analytical techniques described by McCoy (2006). These involved identifying institutional texts and processes that staff referred to when performing work activities; this helped keep the analytical focus on the institutional practices that co-ordinated what happened (McCoy 2006; p 109–110). Maintaining the analytical focus on institutional practices acknowledges that while the people, animals, and physical environment in shelters may be different, the aim of an IE is to investigate how experiences of these individuals are being organised by generalised institutional processes (Smith 1987; p 187). To commence this institutional analysis, we recorded when staff referred to the digital database, performed standardised activities using checklists, examinations and evaluations, and asked them to describe how information was used to categorise and track the status of animals. As well, we asked them to explain how they co-ordinated their work with others and the decisions they made.

We also used analytical techniques described by Rankin (2017a,b) which include mapping, indexing and writing accounts. We developed a chronology using fieldnotes, documents and interviews to first organise our data about the sequence of work processes and activities involved in Henry’s stay and to track and map how the work related to policies and texts. We then indexed the data (Rankin 2017a) to categorise work processes. Indexing differs from many qualitative analysis techniques that code data to generate themes or interpretations. Indexing preserves the empirical descriptions of work processes, which are then sub- and cross-indexed under practices and what they accomplished. For instance, much of the work staff did was work understood to be “monitoring the animals.” These work practices could be cross-indexed under “moving animals to different kennels based on need.” Moving animals was often carried out in parallel with the need to “make space” for animal intake, or to feature an animal in a “high foot traffic area”, in which case the practices could be indexed under both “intake work” and “adoption work.”

Finally, we wrote accounts based on observations and discussions with staff about Henry and other animals with long shelter stays. Accounts included references to the texts people mentioned. We used these accounts from various people and our chronological map to write a full ethnographic account of Henry’s stay.

## Results

### Ethnographic account

The account below is based on observations of staff and their work with Henry – a dog with behavioural problems and a long shelter stay. This account shows that although frontline staff expressed concern about Henry’s difficulties, their work activities are organised mostly to manage the population of animals in the shelter and to promote efficient adoption of most animals, whereas the special needs of animals such as Henry may not be well met.

*I met Henry on June 30, a few days after he was transferred from another shelter branch. Henry had been seized from a property by animal protection officers approximately three weeks earlier. He and six other dogs had been living in unsanitary conditions with a lack of social contact with humans. Notes made by staff on Henry’s file indicate that he seemed anxious and was observed performing behaviours indicative of stress, such as barking at people as they passed his kennel. In a formal behavioural evaluation and review of intake documents to gather information about Henry, staff categorised Henry as an ‘orange dog’, meaning that he has specific behaviours that need to be managed before adoption. A clipboard attached to the front of Henry’s kennel held forms and checklists that staff and volunteers used to record observations. With Henry categorised as a dog with behavioural problems, staff monitored Henry’s behaviour and discussed his progress in meetings. They also did some work with Henry on his behavioural problems and provided written notes to volunteers about how they could help, for example, by using treats to redirect Henry’s attention when he sees another dog, spending extra time with Henry in his kennel after walks, and that he should only be walked by confident volunteers. Observations recorded by volunteers were mixed but mostly positive, with notes like “great walk!”, “sweet boy!”, “no concerns” and a few notes like “difficulty focusing after seeing another dog.”*

*On July 5, during the daily meeting, staff discussed tasks that needed to be completed that day. In addition to regular feeding, walking and cleaning duties, tasks included a behavioural evaluation for a newly arrived dog and scheduling veterinary appointments for two other dogs. They then discussed Henry. Staff were concerned because Henry had been in shelter care for 32 days, much longer than the 11-day average. Staff believed Henry was adoptable and just needed to find an adopter who could help him manage his behaviour, but they were also worried that Henry was becoming increasingly frustrated in the shelter. They made plans to move Henry to the other side of the shelter where there is less human foot traffic, and they discussed featuring Henry online or on social media to help attract an adopter.*

*On July 12, a volunteer told me excitedly that Henry had an adoption application but the next day I learned that the application “fell through.” As days passed, staff noted that it was becoming increasingly difficult for Henry to redirect his focus after seeing dogs on walks; some volunteers indicated that they were no longer comfortable walking Henry. On August 2, 60 days after being seized, shelter administrators decided to euthanase Henry because his reactivity to other dogs was too severe for him to live safely in the community and his welfare in the shelter was deteriorating. During the morning meeting on August 3, staff took a moment to express their sadness about Henry and that they were unable to find him a home. Staff then turned their attention to the list of tasks to complete that day.*

This account is likely familiar to people who work in shelters. How Henry’s life in the shelter unfolded generates questions about how staff spend their time and what directs the day-to-day events in the shelter. The ethnographic account references evaluations and texts and identifies work involved with adoption and behavioural modification and management. It also shows that staff are concerned when a dog has a long stay followed by euthanasia. Thus, although this account is the story of an individual dog, it provides an entry-point to explore a series of tensions that arise in the everyday work of staff in managing animals like Henry. The tensions centre around: conducting behavioural evaluations and assessments, carrying out behavioural modification, and monitoring and anticipating kennel use.

### Conducting behavioural evaluations and assessments

*From the account: In a formal behavioural evaluation and review of intake documents to gather information about Henry, staff categorised Henry as an ‘orange dog’, meaning that he has specific behaviours that need to be managed before adoption.*

Like most physically healthy dogs over five months of age, Henry underwent a standardised behavioural evaluation in the shelter after receiving a medical intake examination. The evaluation uses terms and metrics (standardised across shelters) to categorise dogs based on aggression toward people, aggression toward dogs, excitability, fearfulness, and anxiety when left alone. These metrics correspond with a colour category (other behavioural evaluations use numbers) that designates the dog’s “suitability for rehoming.” Colours include ‘green’, ‘yellow’, ‘orange’ and ‘red.’ A ‘green’ dog is suited to a ‘lifestyle match’ (e.g. a young, athletic dog would be matched with a person who enjoys walking or hiking). A ‘yellow’ dog requires moderate behavioural rehabilitation in shelter and continued management by their adopter, and an ‘orange’ dog requires behaviour modification and evaluation of progress before adoption. A ‘red’ dog may pose a high safety risk to people and thus more information is needed about the animal before a decision about adoptability can be made.

One day, I observed staff member Blake perform an evaluation. Blake explained:

*“The evaluation helps us know about special considerations for placing the dog in a type of home. But I don’t want to put up too many barriers for this dog to getting adopted, and we want to decrease barriers for people who want to adopt animals.”*

To Blake, this work is about finding a balance between providing information to potential adopters without putting up too many “barriers” to adoption. When Blake started work at the shelter, they received training on the Humane Society of the United States ‘Adopters Welcome’ programme (HSUS 2022). This programme aims to facilitate adoption by avoiding specific requirements (e.g. fencing) and promoting a conversational approach to adoption, thus encouraging adopters to see the shelter as a source of support and information.

A few weeks earlier during Henry’s evaluation and the ongoing assessment process including observations of Henry’s behaviour and review of intake documents, Blake identified a few “special considerations”: Henry’s reactivity to other dogs, strong prey drive (he fixates on and tries to chase small animals like squirrels) and separation anxiety (he barks, whines and seems anxious when left alone). Thus, on Henry’s online profile, which was available to people interested in adoption, Blake included that Henry “needs experienced and understanding owners” and ticked boxes next to “no cats” and “no dogs.” These considerations require specific matching and are potential barriers to Henry being adopted quickly. To find “experienced and understanding owners”, staff pay attention to sections of the adoption application regarding previous dog ownership and “problems you (adopters) are willing to work on.” Thus, for staff, following these stipulations to protect the safety of humans and other animals made finding an adopter for Henry a potentially more difficult and lengthy process compared to dogs with few or no “special considerations.”

The work of behavioural evaluations also provides a record of the animal’s behaviour that could be referenced and used to make decisions about animals if there is an incident in the shelter or after adoption. As further safety measures, volunteers are not allowed to interact with dogs until they are assessed by a trained staff member and dogs with dog reactivity or mouthy or jumpy behaviours that could unintentionally harm a human must be handled only by staff or certain experienced volunteers designated by staff. Finally, behavioural evaluation records, medical examination documents and all observation forms used to track an animal’s behaviour while in shelter are kept for seven years and could be reviewed if the animal were to be adopted and then returned or in the case of a future incident with the dog (e.g. harming another animal) after adoption.

A behavioural evaluation takes approximately 30 min to gather materials, complete the evaluation and enter information into the database. In cases where a dog has physical or behavioural problems (e.g. health concerns, cannot be handled safely) staff perform an informal evaluation by observing the animal for their first few days in shelter, reviewing intake documents, and then discussing with managers to determine whether the dog is adoptable and special considerations for potential adopters to know about. This assessment process (a formal or informal evaluation as well as observations and document review) must take place before adoption. This process was reflected when staff member Taylor performed an evaluation for a recent arrival. Taylor explained:

*“We have a potential adopter for this dog, but we don’t want to adopt an adult dog without having done an evaluation, so we at least have something on record about the behaviours we saw.”*

Thus, conducting an evaluation was a requirement even though Taylor knew through informal observations and discussions with staff in advance that the dog was an extremely friendly ‘green’ dog, and indeed, performing the evaluation confirmed what Taylor already knew.

In summary, conducting behavioural evaluations for dogs is part of the social organisation of shelter work. It uses standardised criteria and terms that are used by staff across all shelters. The evaluation and assessment process is specifically linked to three main institutional priorities. These are to identify problem behaviours that would be important for a potential adopter to know; to protect volunteers and community members from potential harm and thereby protect the organisation from legal action; *and* to determine what dogs need (i.e. management, behavioural modification) during their shelter stay and adoption matching. These priorities limit who (i.e. staff, volunteers) is qualified to socialise with exceptional animals like Henry and may lengthen the time needed to find an adopter.

### Carrying out behavioural modification

*From the account: With Henry categorised as a dog with behavioural problems, staff monitored Henry’s behaviour and discussed his progress in meetings.*

It was because of Henry’s separation anxiety, reactivity to other dogs and staff’s review of intake documents that he was categorised through the assessment process as ‘orange.’ According to information on the behavioural evaluation form, ‘orange’ dogs cannot be adopted until “sufficient and appropriate behaviour modification is provided at the shelter or in foster” so that the adopter can continue treatment after adoption, and the dog “must not pose a risk to people or other animals.” These quotes illustrate how the assessment is connected to determining the type of care animals need while in shelter and ensuring safety for humans and animals.

The behavioural evaluation is connected to an institutional text used by many animal shelters in North America: the Asilomar Accords (2004). The Asilomar Accords (AA) were developed by animal sheltering representatives in the USA and include standardised criteria for categorising animals based on their physical and behavioural health. Animals can be categorised as: Healthy, Treatable-Rehabilitatable, Treatable-Manageable or Unhealthy-Untreatable. The AA Adoptability Guidelines were developed to provide additional details about categorising animals and integrated the behavioural evaluation into the health-based criteria (Gordon 2016). Staff consult the AA Adoptability Guidelines when questions arise regarding the adoptability of animals. For ‘orange’ dogs specifically with “aggression towards dogs”, the guidelines state:

*“Dogs assessed orange require in-shelter behaviour modification and evaluation of progress before adoption…* [and] *need to be managed. Decision to provide behaviour modification depends on Society’s ability to match needed resources with problem, for example, foster with knowledge, experience, and/or trainer with expertise.”*

Thus, through the process of categorising Henry via the assessment, and according to the behavioural evaluations used in BC SPCA shelters which are aligned with the AA Adoptability Guidelines and the original AA, Henry was categorised as ‘Treatable-Manageable’ and is thus a dog that needs to be managed.

But for dogs categorised orange, how is behavioural modification and management actually accomplished in everyday work? What staff called “doing behavioural modification” or “BMOD” included a variety of activities that seemed to be added onto their formal responsibilities. In Henry’s case, staff and volunteers used “counter-conditioning” where in cases when Henry saw a dog from a distance on a walk (exposure to a low-level of the stimulus), he was given a treat (receive a reward), which aimed to change Henry’s typical reaction of barking and lunging at other dogs. Staff and volunteers also avoided other dogs on walks, moved other shelter dogs to the back of their kennels before Henry was walked, and spent time sitting with Henry in his kennel during their breaks as he appeared to enjoy being around people. Interactions were recorded and discussed by staff in meetings. Thus, “doing behavioural modification” and managing animals involved multiple activities that required time but seemed to be carried out mostly when staff found extra time.

Staff members Reese and Avery described how they use their time and see their work as connected to adoptability and euthanasia decisions. The quotes express the demands embedded in the staff’s work and the pressures they feel both to accomplish formal and ethical responsibilities for animals in their care. Reese explained:

*“*[What] *if I don’t have enough time, let’s say, to spend with that animal. Knowing the shelter environment is more stressful, if only I had a foster, if only I had more volunteers, I feel stretched thin in a sense of, I still have to do the dishes, but if I don’t spend enough time with* [animal name]*, and she doesn’t make improvements soon a decision* [to euthanase] *might have to get made.”*

Avery added:

*“Euthanasia for behaviour is a grey area. If we get a fearful cat, the shelter administrators might say, ‘let’s see what happens in a few days’, but I know what that means. I need to get this cat to like people in three days! I would love not to feel like that. I will spend my lunch break with the cat to make it adoptable.”*

In the quotes, staff expressed their feeling of responsibility to find time to make animals “adoptable.” Even customer service staff, although delegated to working with human clients, often used spare time to work with animals. And although staff know that there is no specific limit for how long animals can remain at the shelter, staff were aware that long stays reduce the potential for adoption and could make euthanasia more likely.

Shelter administrators expressed a parallel concern about overall capacity, noting in a focus group that not all shelters have the staff resources to perform behavioural modification. Administrators also noted that more animals arriving at shelters today are “harder” animals, meaning those with behavioural or veterinary problems in need of rehabilitation. Administrators also discussed behaviour modification as a specialised task which is the treatment of a behavioural condition; a task that requires training, and they noted that behaviour modification plans, which instruct staff and volunteers on how to work with specific animals, are developed by trained individuals. This designation of behaviour modification as specialised work is important however it is also important to acknowledge the everyday interactions (e.g. feeding, socialising, counter-conditioning) that staff have with animals and their feelings of responsibility toward those animals. These interactions can be considered local knowledge, which in IE includes knowledge and experiential understanding about what the everyday work actually requires; those many activities that job descriptions cannot accommodate but are necessary for the job to get done. In this research into animal sheltering, such knowledge is important to describe because it is integral to the staff’s daily interactions with the shelter animals.

In summary, the tensions that arise in behavioural modification work are rooted in institutional policies and ideas about how staff spend their time, who has training and expertise to do behavioural modification, and how to mitigate risk to people and animals. These ideas organise how tasks are prioritised and how risks, for example those involving working with an animal with behavioural problems, are formulated and addressed. Such tensions have consequences for animals in need of behavioural modification. Moreover, staff feel implicated in, but have little control over, euthanasia decisions for animals with behavioural problems. This is an emotional cost to staff as they bond with and work with these animals daily and feel pressure to find time to do what they can to make them adoptable.

### Monitoring and anticipating kennel use

*From the account: During the daily meeting, staff discussed tasks that needed to be completed that day. In addition to regular feeding, walking and cleaning duties, tasks included a behavioural evaluation for a newly arrived dog and scheduling veterinary appointments for two other dogs.*

Staff performed many tasks related to what they called the “flow” of animals coming in and out of the shelter daily. These tasks include monitoring kennel use in their own shelters and across shelters in the province and anticipating and preparing for the arrival of animals from the community.

Monitoring kennel use involved moving animals within the shelter to accommodate special needs. Staff member Reese explained the difficulty of accommodating the number and various needs of the cats within the shelter:

*“I’m trying to figure out where to put cats all the time. We have anxious cats, sick cats, fearful cats. There is no perfect solution because cats are coming and going all the time.”*

In explaining this work Reese used the term “inventory” to refer to all the cats in the shelter, suggesting that they see the work as managing cats at the population level. Reese explained that they check the database every day to confirm the inventory (called the “In-care inventory” report in shelter software) because cats are “coming and going all the time.” This work includes discussing with other staff, volunteers and administrators about how to move animals within the shelter based on their health and welfare needs, as well as cleaning, sanitising and preparing kennels for the animals.

Although there are fewer dogs than cats in Canadian animal shelters (BC SPCA 2022), the work of monitoring dog kennels required staff to move dogs within the shelter to accommodate their welfare needs. For example, when Henry was in the shelter, another dog (Sam) was quarantined for upper respiratory infection. To limit the spread of infection, staff kennelled Sam away from the other dogs. During one meeting, staff member Blake strategised about kennel use much as Reese had worked to “figure out” where to put cats:

*“Henry’s pretty stressed out with the other dogs in. It will be nice to get Sam out* [adopted after quarantine lifts] *because I want to move Henry on the other side* [of the shelter where there is less foot traffic]*. Every time the other dogs bark it just gets him really stressed out.”*

These examples show the local knowledge possessed by Reese and Blake about the shelter population, individual animal needs, kennels, strategies for managing “foot traffic” and adoptability.

Each week staff also prepare for the arrival of animals transferred from other shelters with the goal of moving animals from rural to urban areas where there will be more potential adopters. Waiting for the transfer to arrive one day, staff member Taylor explained:

*“Any of our animals* [i.e. animals in BC SPCA shelters across the province] *that hit the transfer list go to the bigger shelters that get lots of foot traffic. These animals come in one day on a transfer and then are out* [adopted] *the next day. So we’re moving those animals out and they help to keep our length of stay down.”*

Successful adoption through transfers requires that receiving shelters have available kennels to house animals. Thus, Henry and other long-stay animals limit the number of other animals that can be received on transfer.

Staff also anticipate how much kennel space is needed for animals not yet in shelter care. This work is organised by legal and standardised work practices that vary depending on the type of animal. Regarding ‘stray’ cats, staff frequently answer calls, emails or in-person visits from members of the public who find cats ‘straying.’ Staff explain that municipal animal control laws typically allow cats to roam freely; therefore, the person should try to determine if the cat is owned before bringing it to the shelter. Even so, each month administrators in shelters that accept stray cats calculate the number of empty kennels needed for stray cats based on the monthly average over the previous three years. In many locations stray dogs are the responsibility of the local municipalities. Thus, callers who contact the shelter about stray dogs are usually, depending on whether shelters hold municipal animal control contracts, referred to municipal animal control. Staff refer people to the BC SPCA Animal Helpline if the animals’ condition meets the legal definition of “distress.” Finally, when animal protection officers anticipate seizing animals because they have been found to be in distress, they telephone, email, or speak to shelter administrators and staff to determine where the seized animals could be kenneled.

Standardised practices also organise interactions with people wishing to relinquish an animal. For example, staff member Riley followed a set of standardised work processes when speaking on the telephone with a person wishing to relinquish a cat. After asking for basic information about the cat (age, sex), Riley told the caller that the shelter currently did not have space to house the animal, but they would email the person “owner surrender forms” to fill out. Riley advised the person that they were added to a waiting list and would be contacted when a kennel was available. Later, in a staff meeting, Riley updated the staff and the shelter administrator about the cat and the other animals on the waiting list. Staff follow these same steps for each relinquishment request.

In interacting with the public, customer service staff make decisions based on fairly standardised institutional criteria for kennel use. Staff member Avery explained their understanding of these priorities and noted that one of the most difficult parts of this work is talking to people who are planning to relinquish their animal. They said:

*“We have to prioritise which animals come in first. First, it’s cruelty or neglect cases, then strays and animal control contracts if we have them, then emergency boards, and finally surrenders.”*

Here, Avery expresses one form of knowledge: how the institution prioritises different categories of animals coming into the shelter. They then added:

*“It’s really hard. There are a lot of people in need, sometimes crying on the phone because they need to surrender their animal and I am trying to make it work. But I have to be conscious about the number of animals we have in care following C4C.”*

Here, Avery expresses another form of knowledge: how the institutional priorities create a challenge in their work with people in difficult situations. This creates a tension between these two types of knowledge. One involves adherence to institutional priorities that aim to address animal and community welfare (i.e. animals involved in cruelty investigations require safety that a shelter can provide). The other form of knowledge is concerned with the hard work of talking and responding to people in difficulty.

Avery also references an obligation to “follow C4C” or Capacity for Care. The everyday work practices described earlier also align with other C4C goals: Blake’s work with the behavioural evaluation aimed to minimise barriers to adoption and Blake and Reese’s work monitoring kennel use aimed to improve the welfare of animals by housing them based on their needs. One of the key management goals of C4C is to maintain a low average LOS for targeted animal groups (dogs, puppies, cats, kittens and rabbits). However, tracking average LOS for animal groups does not provide a comprehensive view of the everyday work staff do to deal with atypical animals like Henry or others with long stays in the shelter.

In summary, the routine work of monitoring and anticipating animals requires staff to optimise kennel use based on animal needs and to anticipate and prepare for animals arriving at the shelter. This work also involves assisting members of the public with their questions about stray animals, relinquishment and adoption. Many of these work activities and accompanying texts are linked to C4C. However, these demands on staff time (monitoring kennel use and animal “flow”, receiving transfers, dealing with distressed owners), required frontline staff’s attention to focus on providing basic care for animals in shelter and to prepare for animals that may soon be in shelter care. This led to staff feeling pressure to find time to work with harder animals that have extended shelter stays and require additional care.

## Discussion

Animals such as Henry with behavioural problems face complicated and unstable pathways to adoption that arise as a series of tensions in the everyday work of staff. Our findings do not undermine the C4C approach, but rather describe the everyday work frontline shelter staff do and highlight how this work is connected to institutionalised processes and policies linked to C4C. Exceptional animals like Henry can become somewhat marginalised within work activities designed for other priorities such as providing daily care for animals and anticipating the arrival of animals from the community. These tasks and priorities likely achieve good welfare outcomes for most animals, but they leave insufficient time for staff to work with animals that need additional support before they can be successfully adopted.

The work of staff in conducting dog behavioural evaluations is a standardised, textual work process that helps facilitate successful matching of dogs to adopters and mitigate risks to the broader community. Our study provides insight into how staff understand the work of conducting evaluations. Most of the literature on behavioural evaluations is focused on evaluating their validity (e.g. Duffy *et al.* 2014; Menchetti *et al.* 2019), debating their utility (Patronek & Bradley 2016; Clay *et al.* 2020; see Halm 2021 for cat behavioural evaluations), using evaluations to predict LOS (McGuire *et al.* 2021) or reviewing the type of information gathered about dogs through evaluations (Griffin *et al.* 2022). Little research has been completed with staff who perform evaluations, and although Mornement *et al.* (2010) provided some insights, research is needed to highlight the knowledge and perspectives of staff members who regularly perform evaluations, especially given the common use of dog behavioural evaluations in shelters (Griffin *et al.* 2022).

The everyday work that staff do to rehabilitate problem behaviours has not been well described in the literature even though behavioural problems are a major reason for relinquishment of cats and dogs (Weiss *et al.* 2015; Powell *et al.* 2021), and animals brought from some situations, such as hoarding (McMillan *et al.* 2016) or neglect (Koralesky *et al.* 2023b), may require substantial rehabilitation. Existing research about carrying out behavioural rehabilitation in shelters includes work with inter-dog aggression (Orihel & Fraser 2008), food guarding (Mohan-Gibbons *et al.* 2012) and hoarded cats (van Haaften 2018). However, most studies do not explore who (shelter staff, trainers, researchers, volunteers) actually performed the rehabilitation nor the time and human resources needed. These details are needed to develop a clear understanding of the resources required for doing behavioural modification.

Behavioural modification is defined by experts as a specialised task that treats a behavioural condition through “the proper application of learning principles and training techniques” (Landsberg *et al.* 2013). Because behavioural modification is viewed in this way, workers such as Reese and Avery do not have time allocated to do behavioural modification, yet as they encounter “harder” animals in their everyday work, they feel they must “fit in” such work amongst their formal responsibilities. In the official running of the shelters, formal behavioural modification plans are developed by trained individuals whereupon staff and volunteers follow these plans. While there are opportunities to provide education for staff about behaviour modification (Lilly *et al.* 2021), there may still be scope for shelters to consider how to designate time and to better integrate the experiential and local knowledge of staff in such work given their daily interactions with animals. This may involve understanding how their daily work proceeds, including planned and unplanned work, and where they find time to fit in remedial work with animals. As well, staff emphasised the importance of foster programmes; hence, expanding these programmes so that behavioural modification can occur outside the shelter should be considered.

Providing educational and training opportunities, while ensuring that behaviour modification is done consistently, may be critically important as a way to respond to those “harder” animals that are becoming more common in shelters. The recognition, incorporation and valuing of local knowledge, for example, would involve listening to and observing frontline staff to see the steps they take while performing daily activities and tasks with animals that contribute to the animals’ becoming better socialised. Future research might also identify existing behavioural modification training and implementation practices being done in shelters so as to identify common approaches and best practices that could serve as models and be adjusted based on shelter context. This is important because shelters vary in design and the shelter environment does not adequately provide for animals’ long-term physical and behavioural needs (Association of Shelter Veterinarians 2022). Insights gained might lead to adjusting staffing levels (although financial constraints may need to be considered) and formal job responsibilities to include work with animals in need of behavioural modification and providing animal behaviour and behaviour modification training and techniques to ensure handling methods are in line with the remedial plan.

Our research showed that staff established strong relationships with long-stay animals. Previous research on shelter staff has focused strongly on how staff feel about and cope with performing euthanasia, often framed through the concept of compassion fatigue (e.g. Arluke 1991; Reeve *et al.* 2005; Anderson *et al.* 2013; Andrukonis & Protopopova 2020) and related concepts such as moral injury (Hoy-Gerlach *et al.* 2021). Our findings shed a somewhat different light on this aspect of shelter work. First, staff enjoyed being around Henry and other long-stay animals, and often saw their potential. This led to stress about “running out of time” to help these animals and also to sadness when time did run out. Second, the practicalities of staff’s work activities, especially with animals in need of behavioural modification, are mostly absent from research about shelter staff. Our analysis sheds light on why staff find it challenging to work with such animals. It also shows that an emphasis on time-based measures such as LOS and efficient adoption may not always reflect the time required to work with exceptional animals. Future research, which might be participatory research involving shelter staff and managers, as well as volunteers, could assess whether and how work designated as behaviour modification may be interconnected with other important relational work between staff and animals, including the daily interactions that staff and volunteers have with animals. As well, research that describes the *process* of making euthanasia decisions, for example, describing the information gathered about animals, use of frameworks like the Asilomar Accords, as well as the individuals involved (Koralesky *et al.* 2023a) might provide insight into how and when euthanasia decisions are made for animals with behavioural problems.

Our study identified how the standardised elements of C4C – aimed at managing populations of animals (in the community and shelter) as efficiently as possible (Newbury & Hurley 2013) – directed staff’s everyday work. To begin, C4C’s focus on keeping LOS low while keeping animal numbers manageable is reflected in Avery’s list of priorities for kennel space, Riley’s monitoring of available kennels, and administrators using predictive calculations to monitor kennel use. As well, Blake’s work with the behavioural evaluation aimed to minimise barriers to adoption and Blake and Reese’s work monitoring kennel use aimed to improve the welfare of animals by housing them based on their needs. However, this work left little time to do remedial work with harder animals.

A short LOS (i.e. not having animals in shelters for too long) is likely good for animal welfare (Protopopova 2016; Wagner *et al.* 2018) and is commonly regarded as a measure of shelter efficiency and performance. For example, the average LOS for groups of animals (e.g. dogs, cats) is often reported in national animal shelter statistics (Humane Canada 2020, 2021). Texts used to track information about animals are also used by administrators to calculate Key Performance Indicators such as LOS to benchmark shelter efficiency and help administrators assess how well the shelter is operating.

Most research about C4C implementation has analysed shelter databases and reported positive outcomes, notably for cats (e.g. decreased LOS; Janke *et al.* 2017; Karsten *et al.* 2017). Complementing such research, Hobson *et al.* (2021b) presented thematic interview findings based on shelter staff experiences of C4C, including (as in our study) challenges with the C4C deferred intake relinquishment process. Like that study, our observations further complement high-level performance indicators by ethnographically describing frontline shelter staff work involved with providing daily care for animals and anticipating the arrival of animals from the community. This work is critical in animal sheltering, however it led to staff feeling pressure and stress to find time to work with “harder” animals that they were also responsible for and felt morally obligated to help. Future research that uses observations might further identify specific shelter management practices that could be adjusted to work more in the interests of animals and the people who care directly for them.

## Animal welfare implications and conclusion

In the organisation we studied, daily work is organised to monitor and manage the population of animals in the shelter, anticipate future demands for kennel space, and facilitate efficient adoption for most animals. These socially organised practices direct frontline staff to spend much of their time in specific tasks including interacting with people, deciding where to house animals, monitoring the shelter database, reviewing adoption applications, and providing daily care for animals. However, this left little time for staff to work closely with exceptional animals that required behavioural modification before they could be adopted, and this in turn became a cause of stress for staff who felt responsible for the outcome.

Avenues for further inquiry and possible improvement include:

- Further observations could focus on how the local knowledge of staff could help inform and refine behavioural evaluations and assessments, including identifying which information obtained from assessments is useful for evaluating animals, and the formal and informal work of behavioural modification and other routine work processes.
- Especially with “harder” animals becoming more common in some shelters, allocating appropriate human resources to these animals could help shelters work in the interests of all animals.
- When animals are identified as needing behavioural modification and do not pose risks to the safety of human and animal community members, fostering could be expanded, time could be allocated so that staff can feel less pressure to help these animals be adopted, and volunteer roles could be expanded should financial constraints arise.
- Future IE work could investigate how and whether different tensions might arise in different BC shelters, and study how cultural and geographical differences organise what happens to animals.
